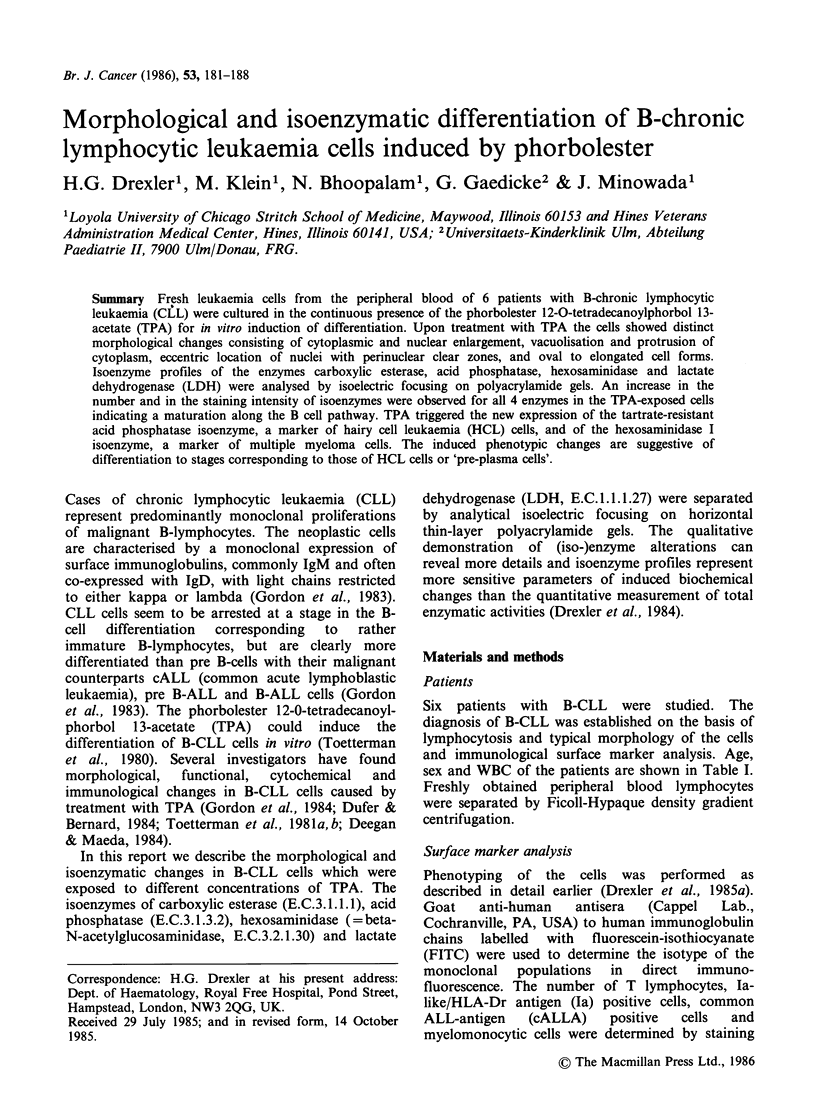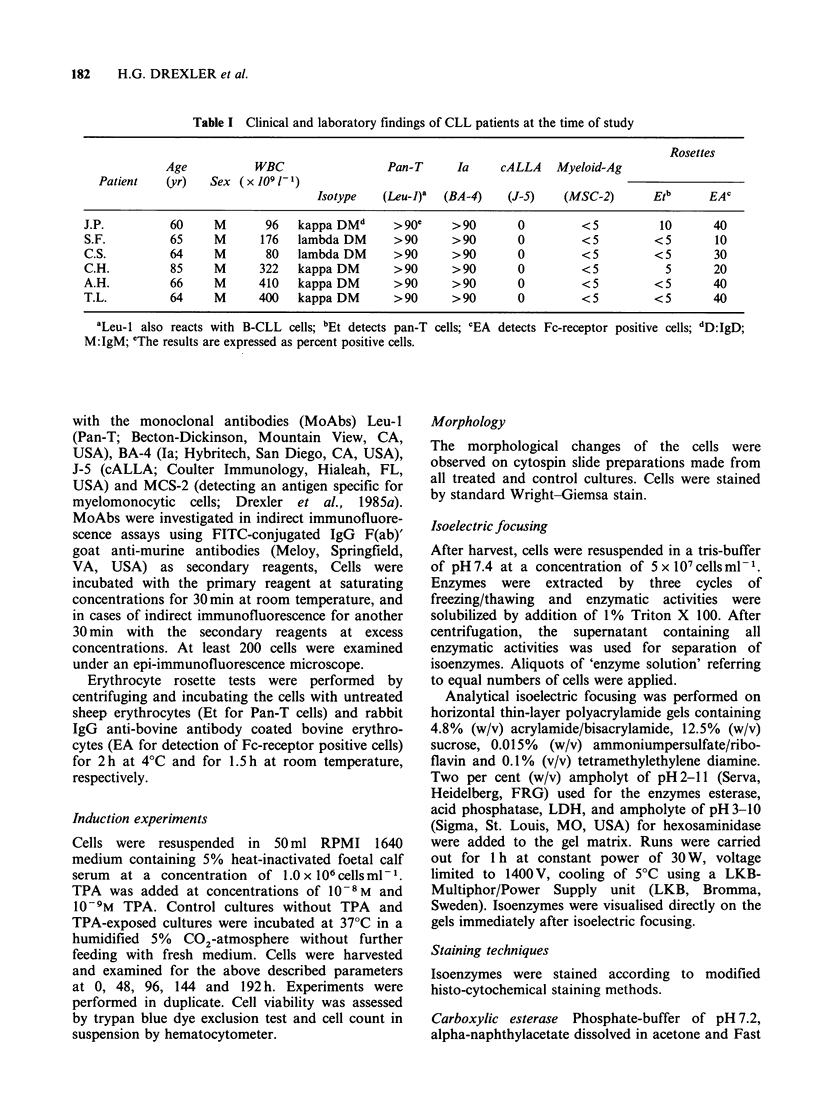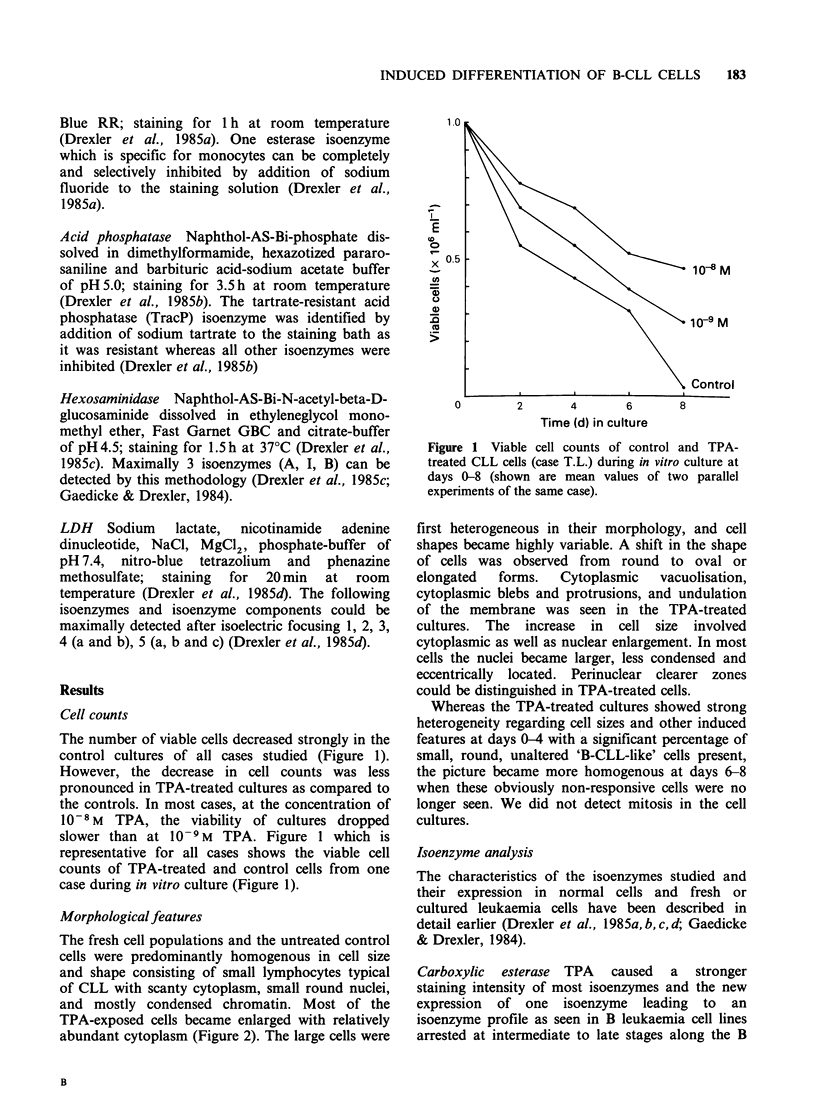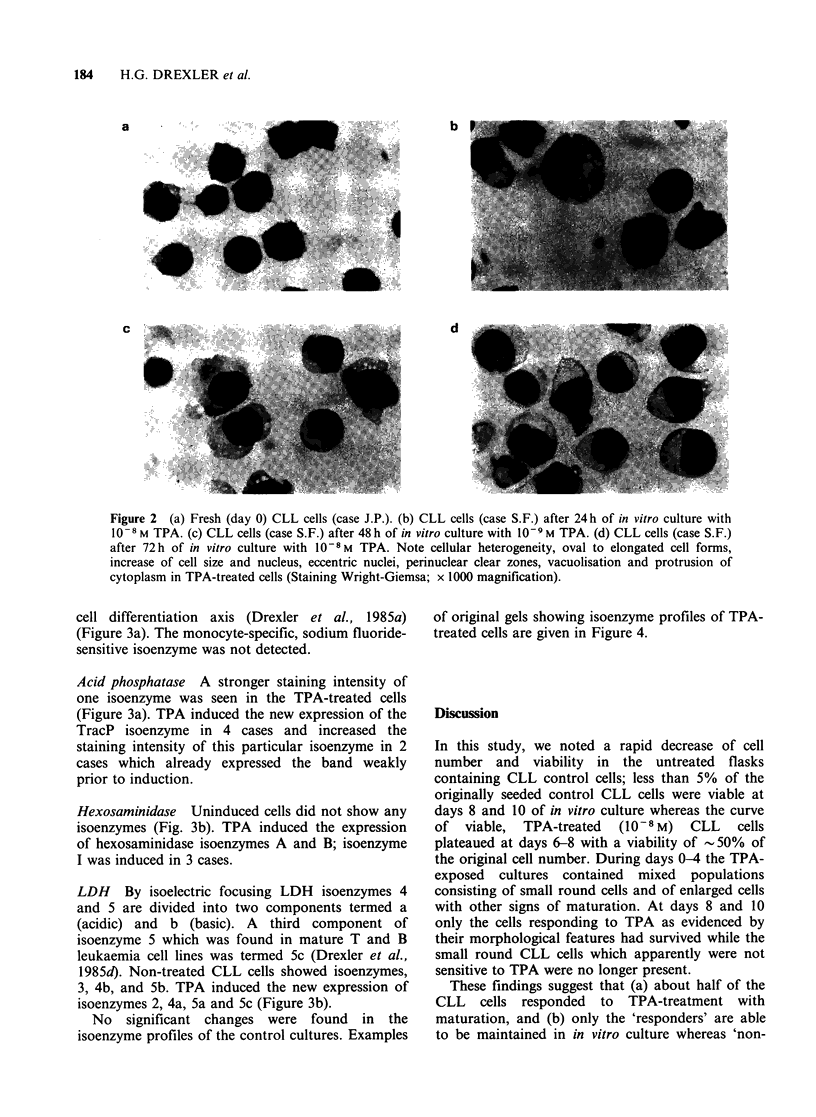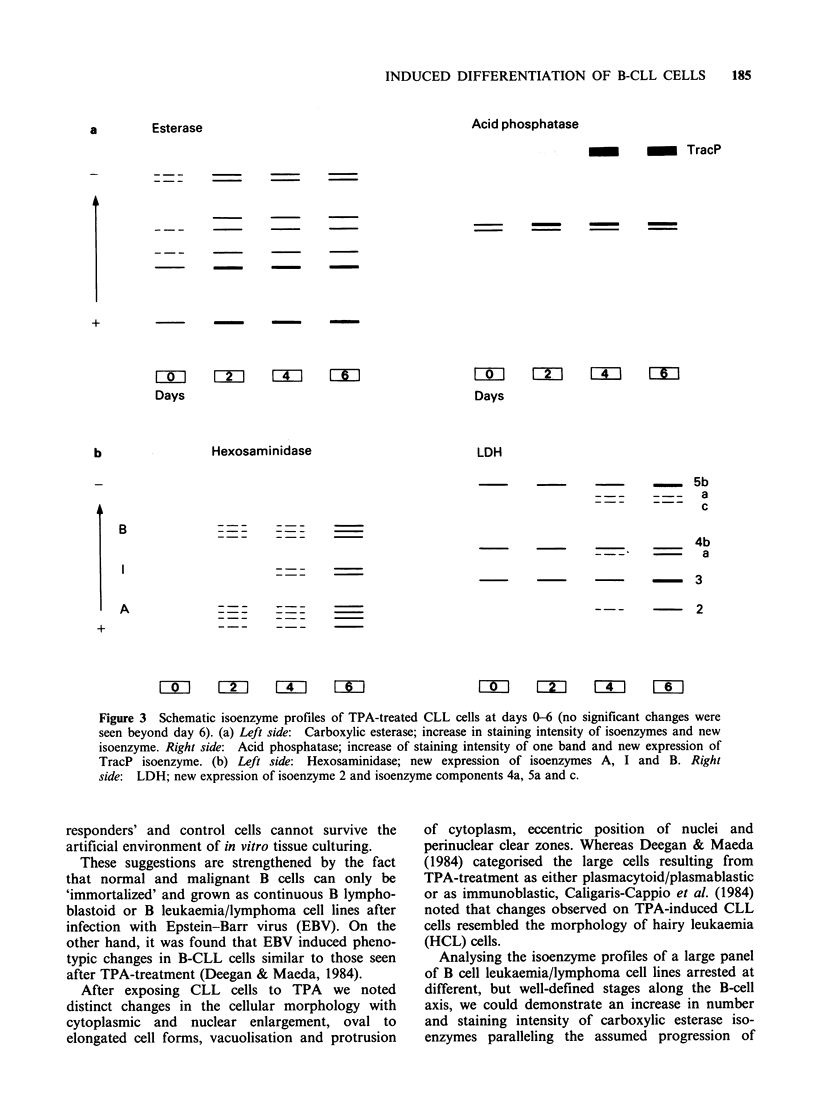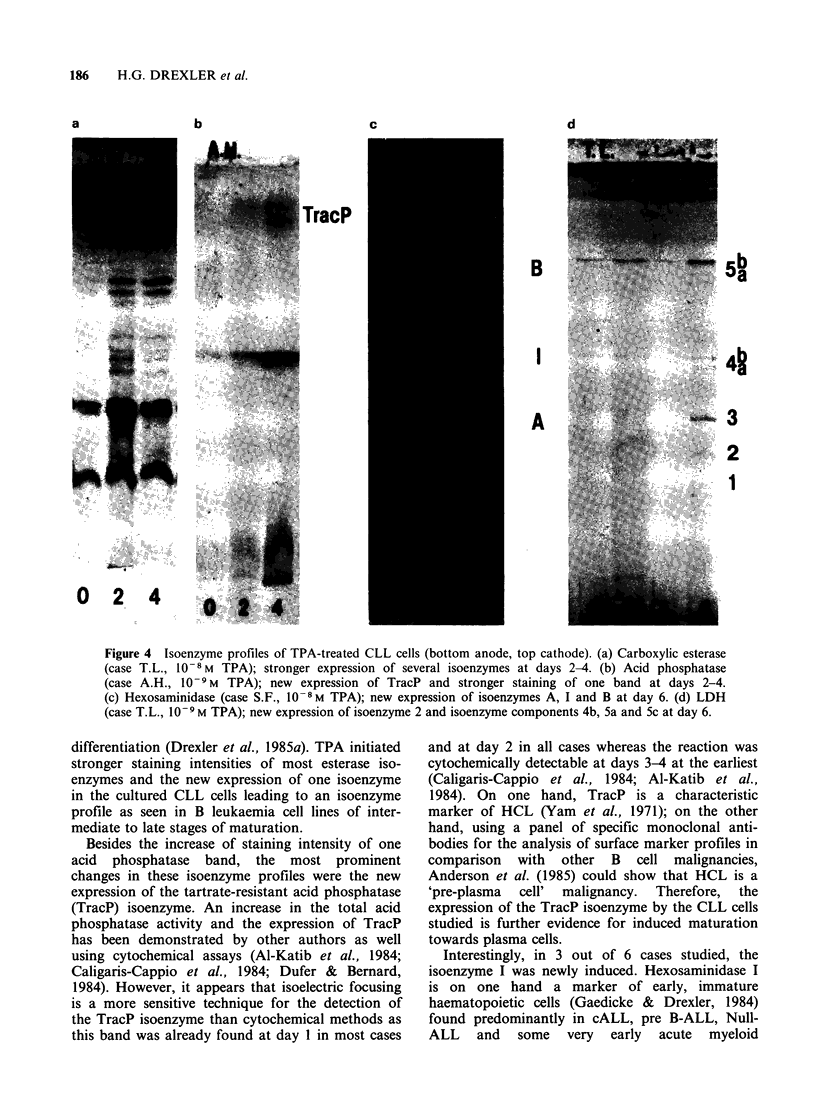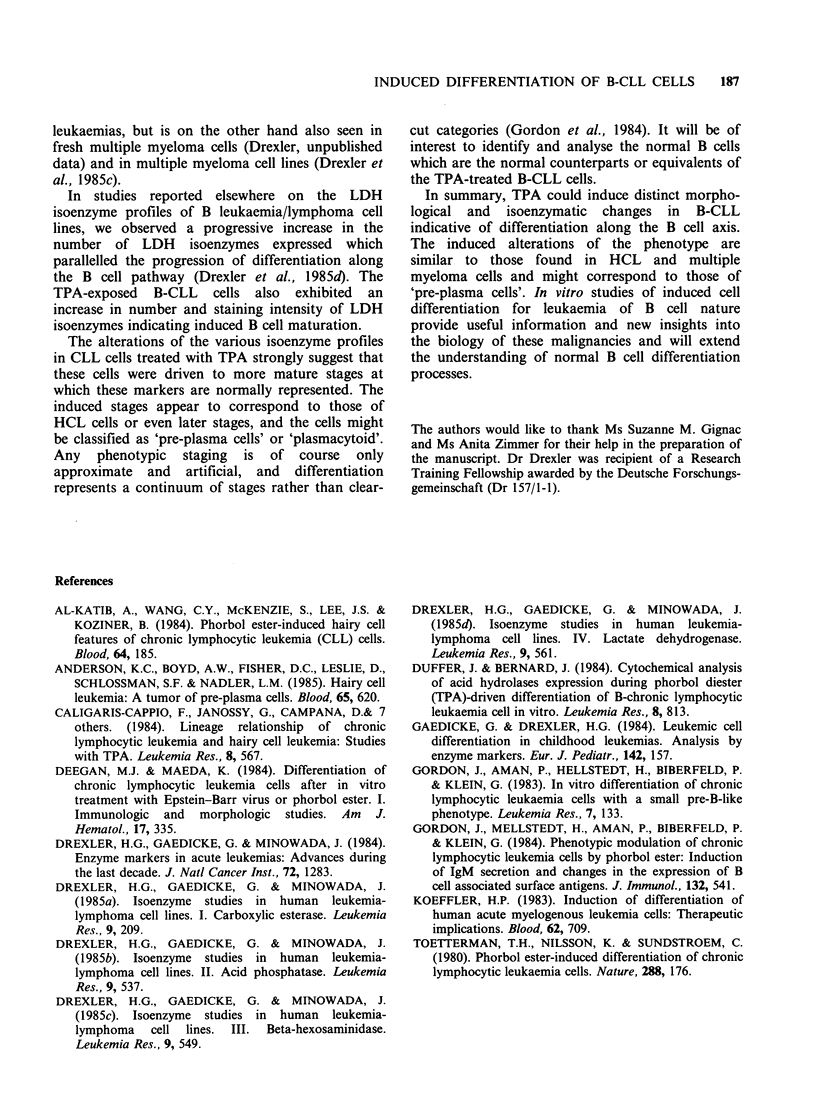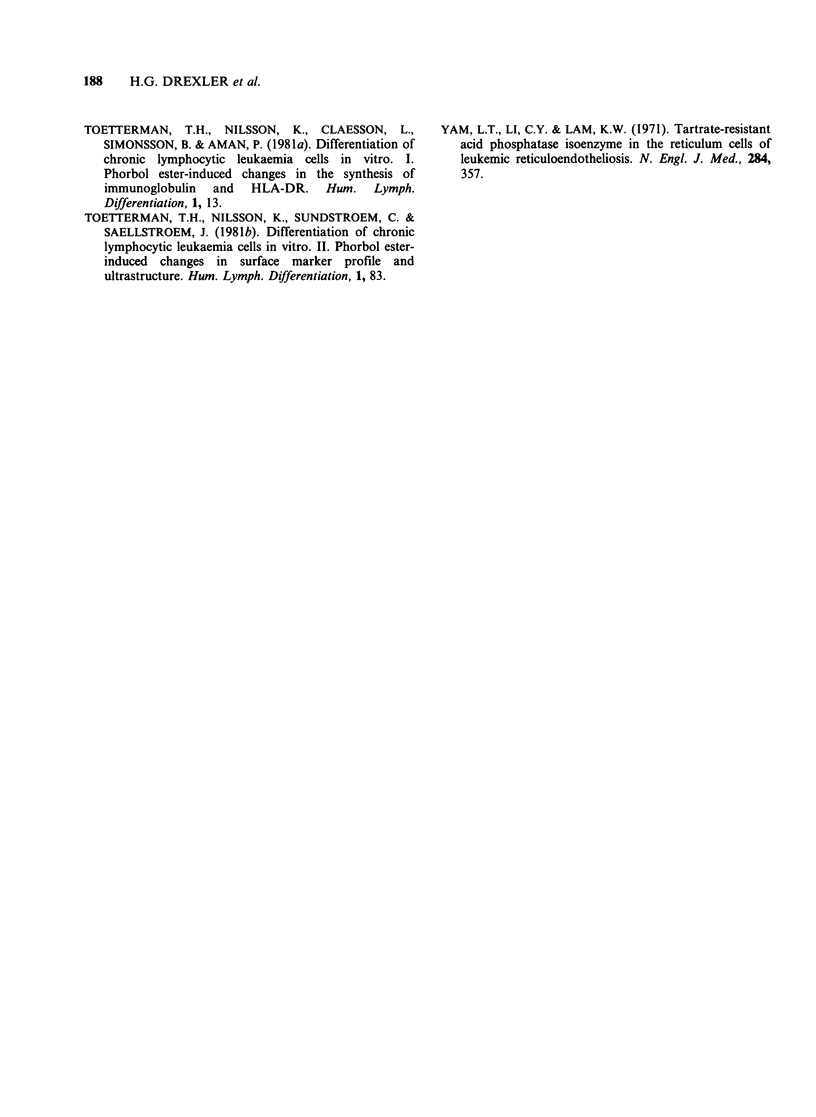# Morphological and isoenzymatic differentiation of B-chronic lymphocytic leukaemia cells induced by phorbolester.

**DOI:** 10.1038/bjc.1986.33

**Published:** 1986-02

**Authors:** H. G. Drexler, M. Klein, N. Bhoopalam, G. Gaedicke, J. Minowada

## Abstract

**Images:**


*Br. J. Cancer* (1986), **53**, 181–188

### Morphological and isoenzymatic differentiation of B-chronic lymphocytic leukaemia cells induced by phorbolester

H.G. Drexler[1], M. Klein[1], N. Bhoopalam[1], G. Gaedicke[2] & J. Minowada[1]

[1]*Loyola University of Chicago Stritch School of Medicine, Maywood, Illinois 60153 and Hines Veterans Administration Medical Center, Hines, Illinois 60141, USA;* [2]*Universitaets-Kinderklinik Ulm, Abteilung Paediatrie II, 7900 Ulm/Donau, FRG.*

**Summary** Fresh leukaemia cells from the peripheral blood of 6 patients with B-chronic lymphocytic leukaemia (CLL) were cultured in the continuous presence of the phorbolester 12-O-tetradecanoylphorbol 13-acetate (TPA) for *in vitro* induction of differentiation. Upon treatment with TPA the cells showed distinct morphological changes consisting of cytoplasmic and nuclear enlargement, vacuolisation and protrusion of cytoplasm, eccentric location of nuclei with perinuclear clear zones, and oval to elongated cell forms. Isoenzyme profiles of the enzymes carboxylic esterase, acid phosphatase, hexosaminidase and lactate dehydrogenase (LDH) were analysed by isoelectric focusing on polyacrylamide gels. An increase in the number and in the staining intensity of isoenzymes were observed for all 4 enzymes in the TPA-exposed cells indicating a maturation along the B cell pathway. TPA triggered the new expression of the tartrate-resistant acid phosphatase isoenzyme, a marker of hairy cell leukaemia (HCL) cells, and of the hexosaminidase I isoenzyme, a marker of multiple myeloma cells. The induced phenotypic changes are suggestive of differentiation to stages corresponding to those of HCL cells or 'pre-plasma cells'.

Cases of chronic lymphocytic leukaemia (CLL) represent predominantly monoclonal proliferations of malignant B-lymphocytes. The neoplastic cells are characterised by a monoclonal expression of surface immunoglobulins, commonly IgM and often co-expressed with IgD, with light chains restricted to either kappa or lambda (Gordon *et al.*, 1983). CLL cells seem to be arrested at a stage in the B-cell differentiation corresponding to rather immature B-lymphocytes, but are clearly more differentiated than pre B-cells with their malignant counterparts cALL (common acute lymphoblastic leukaemia), pre B-ALL and B-ALL cells (Gordon *et al.*, 1983). The phorbolester 12-0-tetradecanoyl-phorbol 13-acetate (TPA) could induce the differentiation of B-CLL cells *in vitro* (Toetterman *et al.*, 1980). Several investigators have found morphological, functional, cytochemical and immunological changes in B-CLL cells caused by treatment with TPA (Gordon *et al.*, 1984; Dufer & Bernard, 1984; Toetterman *et al.*, 1981*a,b*; Deegan & Maeda, 1984).

In this report we describe the morphological and isoenzymatic changes in B-CLL cells which were exposed to different concentrations of TPA. The isoenzymes of carboxylic esterase (E.C.3.1.1.1), acid phosphatase (E.C.3.1.3.2), hexosaminidase (=beta-N-acetylglucosaminidase, E.C.3.2.1.30) and lactate dehydrogenase (LDH, E.C.1.1.1.27) were separated by analytical isoelectric focusing on horizontal thin-layer polyacrylamide gels. The qualitative demonstration of (iso-)enzyme alterations can reveal more details and isoenzyme profiles represent more sensitive parameters of induced biochemical changes than the quantitative measurement of total enzymatic activities (Drexler *et al.*, 1984).

Correspondence: H.G. Drexler at his present address: Dept. of Haematology, Royal Free Hospital, Pond Street, Hampstead, London, NW3 2QG, UK.

Received 29 July 1985; and in revised form, 14 October 1985.

### Materials and methods

### *Patients*

Six patients with B-CLL were studied. The diagnosis of B-CLL was established on the basis of lymphocytosis and typical morphology of the cells and immunological surface marker analysis. Age, sex and WBC of the patients are shown in Table I. Freshly obtained peripheral blood lymphocytes were separated by Ficoll-Hypaque density gradient centrifugation.

### *Surface marker analysis*

Phenotyping of the cells was performed as described in detail earlier (Drexler *et al.*, 1985*a*). Goat anti-human antisera (Cappel Lab., Cochranville, PA, USA) to human immunoglobulin chains labelled with fluorescein-isothiocyanate (FITC) were used to determine the isotype of the monoclonal populations in direct immuno-fluorescence. The number of T lymphocytes, Ia-like/HLA-Dr antigen (Ia) positive cells, common ALL-antigen (cALLA) positive cells and myelomonocytic cells were determined by staining

© The Macmillan Press Ltd., 1986

**Table I** Clinical and laboratory findings of CLL patients at the time of study

| Patient | Age (yr) | Sex | WBC ($\times 10^9 l^{-1}$) | Isotype | Pan-T (Leu-1)[a] | Ia (BA-4) | cALLA (J-5) | Myeloid-Ag (MSC-2) | Rosettes Et[b] | Rosettes EA[c] |
|---|---|---|---|---|---|---|---|---|---|---|
| J.P. | 60 | M | 96 | kappa DM[d] | >90[e] | >90 | 0 | <5 | 10 | 40 |
| S.F. | 65 | M | 176 | lambda DM | >90 | >90 | 0 | <5 | <5 | 10 |
| C.S. | 64 | M | 80 | lambda DM | >90 | >90 | 0 | <5 | <5 | 30 |
| C.H. | 85 | M | 322 | kappa DM | >90 | >90 | 0 | <5 | 5 | 20 |
| A.H. | 66 | M | 410 | kappa DM | >90 | >90 | 0 | <5 | <5 | 40 |
| T.L. | 64 | M | 400 | kappa DM | >90 | >90 | 0 | <5 | <5 | 40 |

[a]Leu-1 also reacts with B-CLL cells; [b]Et detects pan-T cells; [c]EA detects Fc-receptor positive cells; [d]D:IgD; M:IgM; [e]The results are expressed as percent positive cells.

with the monoclonal antibodies (MoAbs) Leu-1 (Pan-T; Becton-Dickinson, Mountain View, CA, USA), BA-4 (Ia; Hybritech, San Diego, CA, USA), J-5 (cALLA; Coulter Immunology, Hialeah, FL, USA) and MCS-2 (detecting an antigen specific for myelomonocytic cells; Drexler *et al.,* 1985*a*). MoAbs were investigated in indirect immunofluorescence assays using FITC-conjugated IgG F(ab)′ goat anti-murine antibodies (Meloy, Springfield, VA, USA) as secondary reagents, Cells were incubated with the primary reagent at saturating concentrations for 30 min at room temperature, and in cases of indirect immunofluorescence for another 30 min with the secondary reagents at excess concentrations. At least 200 cells were examined under an epi-immunofluorescence microscope.

Erythrocyte rosette tests were performed by centrifuging and incubating the cells with untreated sheep erythrocytes (Et for Pan-T cells) and rabbit IgG anti-bovine antibody coated bovine erythrocytes (EA for detection of Fc-receptor positive cells) for 2 h at 4°C and for 1.5 h at room temperature, respectively.

*Induction experiments*

Cells were resuspended in 50 ml RPMI 1640 medium containing 5% heat-inactivated foetal calf serum at a concentration of $1.0 \times 10^6$ cells ml$^{-1}$. TPA was added at concentrations of $10^{-8}$ M and $10^{-9}$ M TPA. Control cultures without TPA and TPA-exposed cultures were incubated at 37°C in a humidified 5% $CO_2$-atmosphere without further feeding with fresh medium. Cells were harvested and examined for the above described parameters at 0, 48, 96, 144 and 192 h. Experiments were performed in duplicate. Cell viability was assessed by trypan blue dye exclusion test and cell count in suspension by hematocytometer.

*Morphology*

The morphological changes of the cells were observed on cytospin slide preparations made from all treated and control cultures. Cells were stained by standard Wright–Giemsa stain.

*Isoelectric focusing*

After harvest, cells were resuspended in a tris-buffer of pH 7.4 at a concentration of $5 \times 10^7$ cells ml$^{-1}$. Enzymes were extracted by three cycles of freezing/thawing and enzymatic activities were solubilized by addition of 1% Triton X 100. After centrifugation, the supernatant containing all enzymatic activities was used for separation of isoenzymes. Aliquots of 'enzyme solution' referring to equal numbers of cells were applied.

Analytical isoelectric focusing was performed on horizontal thin-layer polyacrylamide gels containing 4.8% (w/v) acrylamide/bisacrylamide, 12.5% (w/v) sucrose, 0.015% (w/v) ammoniumpersulfate/riboflavin and 0.1% (v/v) tetramethylethylene diamine. Two per cent (w/v) ampholyt of pH 2–11 (Serva, Heidelberg, FRG) used for the enzymes esterase, acid phosphatase, LDH, and ampholyte of pH 3–10 (Sigma, St. Louis, MO, USA) for hexosaminidase were added to the gel matrix. Runs were carried out for 1 h at constant power of 30 W, voltage limited to 1400 V, cooling of 5°C using a LKB-Multiphor/Power Supply unit (LKB, Bromma, Sweden). Isoenzymes were visualised directly on the gels immediately after isoelectric focusing.

*Staining techniques*

Isoenzymes were stained according to modified histo-cytochemical staining methods.

*Carboxylic esterase* Phosphate-buffer of pH 7.2, alpha-naphthylacetate dissolved in acetone and Fast

Blue RR; staining for 1 h at room temperature (Drexler *et al.*, 1985*a*). One esterase isoenzyme which is specific for monocytes can be completely and selectively inhibited by addition of sodium fluoride to the staining solution (Drexler *et al.*, 1985*a*).

*Acid phosphatase* Naphthol-AS-Bi-phosphate dissolved in dimethylformamide, hexazotized pararosaniline and barbituric acid-sodium acetate buffer of pH 5.0; staining for 3.5 h at room temperature (Drexler *et al.*, 1985*b*). The tartrate-resistant acid phosphatase (TracP) isoenzyme was identified by addition of sodium tartrate to the staining bath as it was resistant whereas all other isoenzymes were inhibited (Drexler *et al.*, 1985*b*)

*Hexosaminidase* Naphthol-AS-Bi-N-acetyl-beta-D-glucosaminide dissolved in ethyleneglycol monomethyl ether, Fast Garnet GBC and citrate-buffer of pH 4.5; staining for 1.5 h at 37°C (Drexler *et al.*, 1985*c*). Maximally 3 isoenzymes (A, I, B) can be detected by this methodology (Drexler *et al.*, 1985*c*; Gaedicke & Drexler, 1984).

*LDH* Sodium lactate, nicotinamide adenine dinucleotide, NaCl, $MgCl_2$, phosphate-buffer of pH 7.4, nitro-blue tetrazolium and phenazine methosulfate; staining for 20 min at room temperature (Drexler *et al.*, 1985*d*). The following isoenzymes and isoenzyme components could be maximally detected after isoelectric focusing 1, 2, 3, 4 (a and b), 5 (a, b and c) (Drexler *et al.*, 1985*d*).

**Figure 1** Viable cell counts of control and TPA-treated CLL cells (case T.L.) during *in vitro* culture at days 0–8 (shown are mean values of two parallel experiments of the same case).

### Results

### Cell counts

The number of viable cells decreased strongly in the control cultures of all cases studied (Figure 1). However, the decrease in cell counts was less pronounced in TPA-treated cultures as compared to the controls. In most cases, at the concentration of $10^{-8}$ M TPA, the viability of cultures dropped slower than at $10^{-9}$ M TPA. Figure 1 which is representative for all cases shows the viable cell counts of TPA-treated and control cells from one case during *in vitro* culture (Figure 1).

### Morphological features

The fresh cell populations and the untreated control cells were predominantly homogenous in cell size and shape consisting of small lymphocytes typical of CLL with scanty cytoplasm, small round nuclei, and mostly condensed chromatin. Most of the TPA-exposed cells became enlarged with relatively abundant cytoplasm (Figure 2). The large cells were first heterogeneous in their morphology, and cell shapes became highly variable. A shift in the shape of cells was observed from round to oval or elongated forms. Cytoplasmic vacuolisation, cytoplasmic blebs and protrusions, and undulation of the membrane was seen in the TPA-treated cultures. The increase in cell size involved cytoplasmic as well as nuclear enlargement. In most cells the nuclei became larger, less condensed and eccentrically located. Perinuclear clearer zones could be distinguished in TPA-treated cells.

Whereas the TPA-treated cultures showed strong heterogeneity regarding cell sizes and other induced features at days 0–4 with a significant percentage of small, round, unaltered 'B-CLL-like' cells present, the picture became more homogenous at days 6–8 when these obviously non-responsive cells were no longer seen. We did not detect mitosis in the cell cultures.

### Isoenzyme analysis

The characteristics of the isoenzymes studied and their expression in normal cells and fresh or cultured leukaemia cells have been described in detail earlier (Drexler *et al.*, 1985*a,b,c,d*; Gaedicke & Drexler, 1984).

*Carboxylic esterase* TPA caused a stronger staining intensity of most isoenzymes and the new expression of one isoenzyme leading to an isoenzyme profile as seen in B leukaemia cell lines arrested at intermediate to late stages along the B

**Figure 2** (a) Fresh (day 0) CLL cells (case J.P.). (b) CLL cells (case S.F.) after 24 h of *in vitro* culture with $10^{-8}$ M TPA. (c) CLL cells (case S.F.) after 48 h of *in vitro* culture with $10^{-9}$ M TPA. (d) CLL cells (case S.F.) after 72 h of *in vitro* culture with $10^{-8}$ M TPA. Note cellular heterogeneity, oval to elongated cell forms, increase of cell size and nucleus, eccentric nuclei, perinuclear clear zones, vacuolisation and protrusion of cytoplasm in TPA-treated cells (Staining Wright-Giemsa; ×1000 magnification).

cell differentiation axis (Drexler *et al.*, 1985*a*) (Figure 3a). The monocyte-specific, sodium fluoride-sensitive isoenzyme was not detected.

*Acid phosphatase* A stronger staining intensity of one isoenzyme was seen in the TPA-treated cells (Figure 3a). TPA induced the new expression of the TracP isoenzyme in 4 cases and increased the staining intensity of this particular isoenzyme in 2 cases which already expressed the band weakly prior to induction.

*Hexosaminidase* Uninduced cells did not show any isoenzymes (Fig. 3b). TPA induced the expression of hexosaminidase isoenzymes A and B; isoenzyme I was induced in 3 cases.

*LDH* By isoelectric focusing LDH isoenzymes 4 and 5 are divided into two components termed a (acidic) and b (basic). A third component of isoenzyme 5 which was found in mature T and B leukaemia cell lines was termed 5c (Drexler *et al.*, 1985*d*). Non-treated CLL cells showed isoenzymes, 3, 4b, and 5b. TPA induced the new expression of isoenzymes 2, 4a, 5a and 5c (Figure 3b).

No significant changes were found in the isoenzyme profiles of the control cultures. Examples of original gels showing isoenzyme profiles of TPA-treated cells are given in Figure 4.

### Discussion

In this study, we noted a rapid decrease of cell number and viability in the untreated flasks containing CLL control cells; less than 5% of the originally seeded control CLL cells were viable at days 8 and 10 of *in vitro* culture whereas the curve of viable, TPA-treated ($10^{-8}$ M) CLL cells plateaued at days 6–8 with a viability of ~50% of the original cell number. During days 0–4 the TPA-exposed cultures contained mixed populations consisting of small round cells and of enlarged cells with other signs of maturation. At days 8 and 10 only the cells responding to TPA as evidenced by their morphological features had survived while the small round CLL cells which apparently were not sensitive to TPA were no longer present.

These findings suggest that (a) about half of the CLL cells responded to TPA-treatment with maturation, and (b) only the 'responders' are able to be maintained in *in vitro* culture whereas 'non-

**Figure 3** Schematic isoenzyme profiles of TPA-treated CLL cells at days 0–6 (no significant changes were seen beyond day 6). (a) *Left side*: Carboxylic esterase; increase in staining intensity of isoenzymes and new isoenzyme. *Right side*: Acid phosphatase; increase of staining intensity of one band and new expression of TracP isoenzyme. (b) *Left side*: Hexosaminidase; new expression of isoenzymes A, I and B. *Right side*: LDH; new expression of isoenzyme 2 and isoenzyme components 4a, 5a and c.

responders' and control cells cannot survive the artificial environment of *in vitro* tissue culturing.

These suggestions are strengthened by the fact that normal and malignant B cells can only be 'immortalized' and grown as continuous B lymphoblastoid or B leukaemia/lymphoma cell lines after infection with Epstein–Barr virus (EBV). On the other hand, it was found that EBV induced phenotypic changes in B-CLL cells similar to those seen after TPA-treatment (Deegan & Maeda, 1984).

After exposing CLL cells to TPA we noted distinct changes in the cellular morphology with cytoplasmic and nuclear enlargement, oval to elongated cell forms, vacuolisation and protrusion of cytoplasm, eccentric position of nuclei and perinuclear clear zones. Whereas Deegan & Maeda (1984) categorised the large cells resulting from TPA-treatment as either plasmacytoid/plasmablastic or as immunoblastic, Caligaris-Cappio *et al.* (1984) noted that changes observed on TPA-induced CLL cells resembled the morphology of hairy leukaemia (HCL) cells.

Analysing the isoenzyme profiles of a large panel of B cell leukaemia/lymphoma cell lines arrested at different, but well-defined stages along the B-cell axis, we could demonstrate an increase in number and staining intensity of carboxylic esterase isoenzymes paralleling the assumed progression of

**Figure 4** Isoenzyme profiles of TPA-treated CLL cells (bottom anode, top cathode). (a) Carboxylic esterase (case T.L., $10^{-8}$ M TPA); stronger expression of several isoenzymes at days 2–4. (b) Acid phosphatase (case A.H., $10^{-9}$ M TPA); new expression of TracP and stronger staining of one band at days 2–4. (c) Hexosaminidase (case S.F., $10^{-8}$ M TPA); new expression of isoenzymes A, I and B at day 6. (d) LDH (case T.L., $10^{-9}$ M TPA); new expression of isoenzyme 2 and isoenzyme components 4b, 5a and 5c at day 6.

differentiation (Drexler *et al.*, 1985*a*). TPA initiated stronger staining intensities of most esterase isoenzymes and the new expression of one isoenzyme in the cultured CLL cells leading to an isoenzyme profile as seen in B leukaemia cell lines of intermediate to late stages of maturation.

Besides the increase of staining intensity of one acid phosphatase band, the most prominent changes in these isoenzyme profiles were the new expression of the tartrate-resistant acid phosphatase (TracP) isoenzyme. An increase in the total acid phosphatase activity and the expression of TracP has been demonstrated by other authors as well using cytochemical assays (Al-Katib *et al.*, 1984; Caligaris-Cappio *et al.*, 1984; Dufer & Bernard, 1984). However, it appears that isoelectric focusing is a more sensitive technique for the detection of the TracP isoenzyme than cytochemical methods as this band was already found at day 1 in most cases and at day 2 in all cases whereas the reaction was cytochemically detectable at days 3–4 at the earliest (Caligaris-Cappio *et al.*, 1984; Al-Katib *et al.*, 1984). On one hand, TracP is a characteristic marker of HCL (Yam *et al.*, 1971); on the other hand, using a panel of specific monoclonal antibodies for the analysis of surface marker profiles in comparison with other B cell malignancies, Anderson *et al.* (1985) could show that HCL is a 'pre-plasma cell' malignancy. Therefore, the expression of the TracP isoenzyme by the CLL cells studied is further evidence for induced maturation towards plasma cells.

Interestingly, in 3 out of 6 cases studied, the isoenzyme I was newly induced. Hexosaminidase I is on one hand a marker of early, immature haematopoietic cells (Gaedicke & Drexler, 1984) found predominantly in cALL, pre B-ALL, Null-ALL and some very early acute myeloid leukaemias, but is on the other hand also seen in fresh multiple myeloma cells (Drexler, unpublished data) and in multiple myeloma cell lines (Drexler *et al.*, 1985*c*).

In studies reported elsewhere on the LDH isoenzyme profiles of B leukaemia/lymphoma cell lines, we observed a progressive increase in the number of LDH isoenzymes expressed which parallelled the progression of differentiation along the B cell pathway (Drexler *et al.*, 1985*d*). The TPA-exposed B-CLL cells also exhibited an increase in number and staining intensity of LDH isoenzymes indicating induced B cell maturation.

The alterations of the various isoenzyme profiles in CLL cells treated with TPA strongly suggest that these cells were driven to more mature stages at which these markers are normally represented. The induced stages appear to correspond to those of HCL cells or even later stages, and the cells might be classified as 'pre-plasma cells' or 'plasmacytoid'. Any phenotypic staging is of course only approximate and artificial, and differentiation represents a continuum of stages rather than clear-cut categories (Gordon *et al.*, 1984). It will be of interest to identify and analyse the normal B cells which are the normal counterparts or equivalents of the TPA-treated B-CLL cells.

In summary, TPA could induce distinct morphological and isoenzymatic changes in B-CLL indicative of differentiation along the B cell axis. The induced alterations of the phenotype are similar to those found in HCL and multiple myeloma cells and might correspond to those of 'pre-plasma cells'. *In vitro* studies of induced cell differentiation for leukaemia of B cell nature provide useful information and new insights into the biology of these malignancies and will extend the understanding of normal B cell differentiation processes.

The authors would like to thank Ms Suzanne M. Gignac and Ms Anita Zimmer for their help in the preparation of the manuscript. Dr Drexler was recipient of a Research Training Fellowship awarded by the Deutsche Forschungsgemeinschaft (Dr 157/1-1).

### References

AL-KATIB, A., WANG, C.Y., McKENZIE, S., LEE, J.S. & KOZINER, B. (1984). Phorbol ester-induced hairy cell features of chronic lymphocytic leukemia (CLL) cells. *Blood*, **64**, 185.

ANDERSON, K.C., BOYD, A.W., FISHER, D.C., LESLIE, D., SCHLOSSMAN, S.F. & NADLER, L.M. (1985). Hairy cell leukemia: A tumor of pre-plasma cells. *Blood*, **65**, 620.

CALIGARIS-CAPPIO, F., JANOSSY, G., CAMPANA, D.& 7 others. (1984). Lineage relationship of chronic lymphocytic leukemia and hairy cell leukemia: Studies with TPA. *Leukemia Res.*, **8**, 567.

DEEGAN, M.J. & MAEDA, K. (1984). Differentiation of chronic lymphocytic leukemia cells after in vitro treatment with Epstein–Barr virus or phorbol ester. I. Immunologic and morphologic studies. *Am J. Hematol.*, **17**, 335.

DREXLER, H.G., GAEDICKE, G. & MINOWADA, J. (1984). Enzyme markers in acute leukemias: Advances during the last decade. *J. Natl Cancer Inst.*, **72**, 1283.

DREXLER, H.G., GAEDICKE, G. & MINOWADA, J. (1985*a*). Isoenzyme studies in human leukemia-lymphoma cell lines. I. Carboxylic esterase. *Leukemia Res.*, **9**, 209.

DREXLER, H.G., GAEDICKE, G. & MINOWADA, J. (1985*b*). Isoenzyme studies in human leukemia-lymphoma cell lines. II. Acid phosphatase. *Leukemia Res.*, **9**, 537.

DREXLER, H.G., GAEDICKE, G. & MINOWADA, J. (1985*c*). Isoenzyme studies in human leukemia-lymphoma cell lines. III. Beta-hexosaminidase. *Leukemia Res.*, **9**, 549.

DREXLER, H.G., GAEDICKE, G. & MINOWADA, J. (1985*d*). Isoenzyme studies in human leukemia-lymphoma cell lines. IV. Lactate dehydrogenase. *Leukemia Res.*, **9**, 561.

DUFFER, J. & BERNARD, J. (1984). Cytochemical analysis of acid hydrolases expression during phorbol diester (TPA)-driven differentiation of B-chronic lymphocytic leukaemia cell in vitro. *Leukemia Res.*, **8**, 813.

GAEDICKE, G. & DREXLER, H.G. (1984). Leukemic cell differentiation in childhood leukemias. Analysis by enzyme markers. *Eur. J. Pediatr.*, **142**, 157.

GORDON, J., AMAN, P., HELLSTEDT, H., BIBERFELD, P. & KLEIN, G. (1983). In vitro differentiation of chronic lymphocytic leukaemia cells with a small pre-B-like phenotype. *Leukemia Res.*, **7**, 133.

GORDON, J., MELLSTEDT, H., AMAN, P., BIBERFELD, P. & KLEIN, G. (1984). Phenotypic modulation of chronic lymphocytic leukemia cells by phorbol ester: Induction of IgM secretion and changes in the expression of B cell associated surface antigens. *J. Immunol.*, **132**, 541.

KOEFFLER, H.P. (1983). Induction of differentiation of human acute myelogenous leukemia cells: Therapeutic implications. *Blood*, **62**, 709.

TOETTERMAN, T.H., NILSSON, K. & SUNDSTROEM, C. (1980). Phorbol ester-induced differentiation of chronic lymphocytic leukaemia cells. *Nature*, **288**, 176.

TOETTERMAN, T.H., NILSSON, K., CLAESSON, L., SIMONSSON, B. & AMAN, P. (1981*a*). Differentiation of chronic lymphocytic leukaemia cells in vitro. I. Phorbol ester-induced changes in the synthesis of immunoglobulin and HLA-DR. *Hum. Lymph. Differentiation*, **1**, 13.

TOETTERMAN, T.H., NILSSON, K., SUNDSTROEM, C. & SAELLSTROEM, J. (1981*b*). Differentiation of chronic lymphocytic leukaemia cells in vitro. II. Phorbol ester-induced changes in surface marker profile and ultrastructure. *Hum. Lymph. Differentiation*, **1**, 83.

YAM, L.T., LI, C.Y. & LAM, K.W. (1971). Tartrate-resistant acid phosphatase isoenzyme in the reticulum cells of leukemic reticuloendotheliosis. *N. Engl. J. Med.*, **284**, 357.